# Non-Coding RNAs Participate in the Pathogenesis of Neuroblastoma

**DOI:** 10.3389/fonc.2021.617362

**Published:** 2021-02-24

**Authors:** Omidvar Rezaei, Kasra Honarmand Tamizkar, Mohammadreza Hajiesmaeili, Mohammad Taheri, Soudeh Ghafouri-Fard

**Affiliations:** ^1^ Skull Base Research Center, Loghman Hakim Hospital, Shahid Beheshti University of Medical Sciences, Tehran, Iran; ^2^ Department of Medical Genetics, Shahid Beheshti University of Medical Sciences, Tehran, Iran; ^3^ Urogenital Stem Cell Research Center, Shahid Beheshti University of Medical Sciences, Tehran, Iran

**Keywords:** miRNA, lncRNA, neuroblastoma, expression, polymorphism

## Abstract

Neuroblastoma is one of the utmost frequent neoplasms during the first year of life. This pediatric cancer is believed to be originated during the embryonic life from the neural crest cells. Previous studies have detected several types of chromosomal aberrations in this tumor. More recent studies have emphasized on expression profiling of neuroblastoma samples to identify the dysregulated genes in this type of cancer. Non-coding RNAs are among the mostly dysregulated genes in this type of cancer. Such dysregulation has been associated with a number of chromosomal aberrations that are frequently detected in neuroblastoma. In this study, we explain the role of non-coding transcripts in the malignant transformation in neuroblastoma and their role as biomarkers for this pediatric cancer.

## Introduction

Neuroblastoma is a neoplasm originated from the neural crest of the sympathetic part of autonomic system (1) during the embryonic life (2). This malignancy is among the most common childhood cancers particularly during the first year of life (3). Neuroblastoma has a heterogeneous course in terms of both pathobiology and clinical manifestations. Several therapeutic options such as surgical removal of the tumor, chemotherapy, radiotherapy, and bone marrow transplantation are being applied for neuroblastoma (4). Spontaneous regression might also happen in the course of neuroblastoma (5). This tumor is associated with several genetic and chromosomal abnormalities that affect its clinical course and prognosis namely *MYCN* amplification, loss of distal portion of chromosome (chr) 1p and gain of 17q (6). Other chromosomal abnormalities detected in neuroblastoma are loss of 11q, 3p, 4p, 9p, 14q, and gain of 1q, 7q, 2p, and 11p (7–9). In addition to these chromosomal aberrations, dysregulation of several genes including non-coding RNAs (ncRNAs) are linked with this cancer (10). These kinds of transcripts have regulatory impact on other genes, hence constructing an epigenetic layer of gene regulation. They are classified based on their sizes to long non-coding RNAs (lncRNAs) and microRNAs (miRNAs) with the former having more than 200 nucleotides and the latter being about 22 nucleotides (11). Based on the speculation stated by the ENCODE consortium regarding the recognition of “biochemical functions for 80% of the genome” (12), ncRNAs have attained much attention during the recent decade particularly in the field of cancer research. In the current study, we explain the role of lncRNAs and miRNAs in the evolution of neuroblastoma and their role as biomarkers for this pediatric cancer.

### Dysregulated miRNAs in Neuroblastoma

Chen and Stallings have measured expression of 157 miRNAs in neuroblastoma samples. They have displayed differential pattern of 32 miRNAs between tumor with favorable prognosis and those with poor prognosis. Notably, several of these miRNAs were down-regulated in neuroblastoma samples harboring *MYCN* amplification, which was associated with unfavorable outcome. Cell line studies have shown the role of retinoic acid in the modulation of expression of miRNAs in a *MYCN*-amplified cell line. Among the dysregulated miRNAs has been miR-184 which participates in the regulation of apoptosis. MYCN might exert its tumorigenic effects *via* modulating expression of miRNAs that participate in neural cell differentiation or apoptotic processes (13).

**Figure 1:**
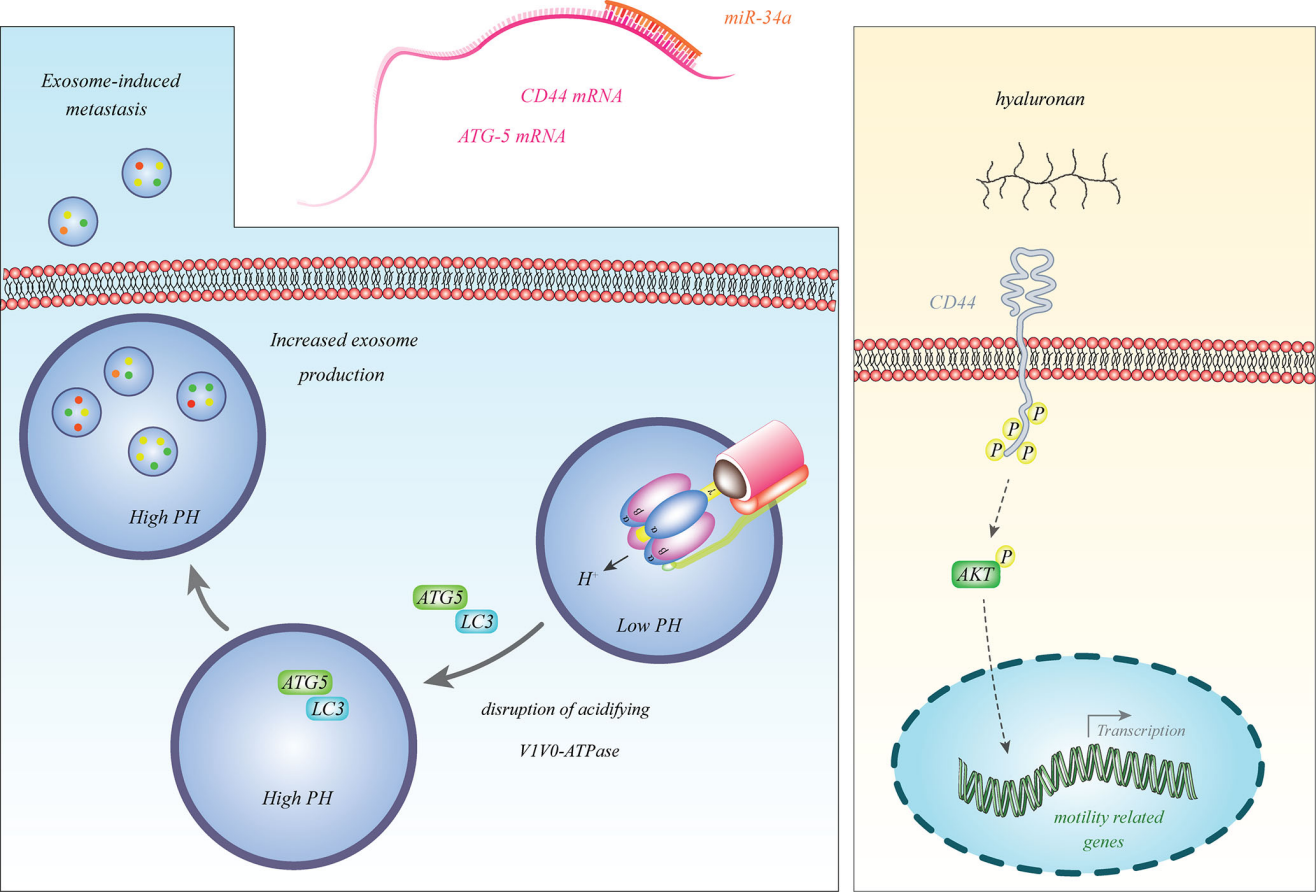
miR-34a binds with the 3’ UTR of ATG5 and CD44 transcripts to reduce their expressions. Decreased expression of miR-34a in neuroblastoma leads to over-expression of ATG5 and CD44 (17, 18). CD44 is a cell surface receptor which can bind with hyaluronan and induce expression of genes that promote progression of cancer (19). ATG5 can dissociate V1V0-ATPase, increase pH in multivesicular bodies and enhance secretion of exosomes to promote cancer metastasis (20).

Among the firstly discovered tumor suppressor miRNAs in neuroblastoma was miR-34a (14), which is transcribed from a frequently deleted region in neuroblastoma i.e. 1p36.23. This miRNA was particularly down-regulated in neuroblastoma samples with 1p deletion (14). Since miR-34a inhibits expression of the E2F3 transcription factor, its down-regulation facilitates cell cycle progression (14). Subsequent studies have also verified the tumor suppressive impact of miR-34a in the neuroblastoma cells and its inhibitory effects on the expression of BCL2 and MYCN (15, 16). miR-34a also binds with the 3’ UTR of ATG5 and CD44 transcripts and decreases their expressions. Down-regulation of miR-34a in neuroblastoma cells results in the over-expression of ATG5 and CD44 (17, 18). CD44 is a cell surface receptor which can bind with hyaluronan and induce expression of genes that promote progression of cancer (19). ATG5 can dissociate V1V0-ATPase, increase pH in multivesicular bodies and enhance secretion of exosomes to facilitate cancer metastasis (20). Thus, miR-34a affects the progression of neuroblastoma through different mechanisms. **Figure 1** shows some aspects of participation of miR-34a in the pathogenesis of neuroblastoma.

miR-542-5p is another tumor suppressor miRNA whose down-regulation in neuroblastoma has conferred poor clinical outcome. Notably, forced up-regulation of miR-542-5p has resulted in attenuation of neuroblastoma invasive properties and tumor growth bot *in vitro* and *in vivo* (21). Moreover, expression of miR-490-5p has been diminished in neuroblastoma tissues and cells. Forced overexpression of miR-490-5p has diminished cell proliferation migration and invasiveness, prompted G0/G1 arrest in cells and induced cell apoptosis. MYEOV has been confirmed to be the target of miR-490-5p through which miR-490-5p blocks neuroblastoma progression (22). **Table 1** recapitulates the results of studies which described down-regulation of miRNAs in neuroblastoma.

**Table 1:** Down-regulated miRNA in neuroblastoma (NB, Neuroblastoma; ANT, adjacent normal tissues; OS, overall survival; EFS, event-free survival).

miRNA	Specimens	Cell line	Targets/regulators	Signaling pathway	Function	Effect of miRNA down-regulation on patient’s prognosis	Reference
miR-490-5p	72 tumor tissues and ANTs	SH-SY5Y, SK-NSH, U343	MYEOV	–	Down-regulated miR-490-5p levels correlate with advanced INSS stage, lymph node involvement, and poor outcome. MiR-490-5p overexpression thwarts cell proliferation, migratory capacities, invasive effects, and enhances the cell cycle arrest and apoptosis.	Poor survival	(22)
miR-144		SH-SY5Y, SK-N-SH, HUVEC	MYCN		miR-144 influences proliferation, apoptosis and cisplatin resistance.		
miR-144-3p	46 pairs of NB ANTs	SK-N-SH, SH-SY5Y, HUVEC,	HOXA7	–	miR-144-3p repression results in the advancement of cell proliferation, cell cycle progression, and cell migration. Down-regulation of miR-144-3p level correlates with advanced tumor stage, greater carcinoma size, and lymph node metastasis.	–	(23)
miR-34a	35 pediatric NB patients, 15 normal adrenal tissue	SH-SY5Y	MMP-2, MMP-14, HNF4α	–	miR-34a down-regulation increases cell proliferation, migration, and invasion.	–	(24)
	18 NB primary and their metastatic tissues	SH-SY5Y, IMR-32	CD44	–	miR-34a repression results in enhanced metastasis, proliferation, and invasion rates in NB cells.	–	(17)
	32 NB and ANTs	SH-SY5Y, SK-N-SH, HUVEC	ATG5	–	proliferation, migration, and invasion rate increase following the miR-34a repression, and the apoptosis rate diminishes.	Lower survival rate	(18)
miR-183	–	IMR-32, SH-SY5Y, SK-N-MC, SK-N-SH, HEK293, KCLB, HEF, SK-N-DZ	-/MYCN, HDAC2	–	MYCN inhibition increases the pro-apoptotic miR-183 levels.	–	(25)
	–	BE(2)-C, Kelly	MCM complex	–	miR-183 down-regulates the MCM complex.	–	(26)
miR-323a-5p	253 NB patients	SK-N-AS, SH-SY5Y, IMR-32, HEK293T141, CHLA-90, SK-N-BE(2), LA1-5	CHAF1A, KIF11, INCENP, CDC25A, CCND1, FADD, E2F2	–	These miRNAs reduce cell proliferation, cell viability, cell cycle, and tumor growth, though they increase the apoptosis rate.	–	(27)
miR-342-5p			AKT2, CCND1, MKNK2, BCLX			Poor OS	
miR-34b	–	SH-SY5Y, IMR-32, KELLY	DLL1	Notch-Delta	miRNA-34b markedly down-regulates the DLL1 mRNA expression levels, arrests cell proliferation, induces neuronal differentiation in malignant NB cells.	–	(28)
miR-145	–	SH-SY5Y	Bnip3	–	miR-145 inhibition promotes mitophagy activity and subsequently increases SH-SY5Y cell survival.	–	(29)
miR-2110	SEQC dataset: 498 NB patients	BE(2)-C, SKNDZ, CHLA-90, SKNFI	TSKU	–	miR-2110 overexpression induces cell differentiation and inhibits cell survival.	Poor OS and EFS	(30)
miR-186	GSE62564 dataset: 498 NB patients	CHLA-136, LAN-5, CHLA-255, HEK293T	MYCN, AURKA, TGFBR1, TGFBR2, TGFβ1	TGFβ	miR-186 lower expression levels relate to a poor prognosis in NB patients that directly correlates with NK activation markers.	Poor EFS and OS	(31)
let-7	–	KELLY, BE2C, SH-SY5Y	TGF-βRI, LMO1, MYCN	–	let-7 decreases the expression levels of TGF-βRI, LMO1, and MYCN.	–	(32)
	–	BE(2)-C, SMS-KCNR, CHLA90	-/DFMO, LIN28B, MYCN	–	Difluoromethylornithine inhibits ornithine decarboxylase, which in turn regulates polyamines. Polyamines regulate eIF-5A, which is a modulator of the LIN28/Let-7 axis. Difluoromethylornithine reduces neurosphere formation, ATP production, and LIN28B and MYCN protein levels yet enhances let-7.	–	(33)
	GSE81500 dataset: 172 NB patients	BE(2)C, PA-1, IMR90, SK-N-AS, SH-SY5Y, HEK293T, SK-N-DZ, Kelly	-/MYCN	–	Genetic loss of let-7 is common in NB and is negatively associated with MYCN amplification. Down-regulation of let-7 is associated with poor outcomes.	Lower OS	(34)
miR-15a/miR-16-1	–	HTLA-230, HTLA-ER, HCT116 TP53−/−	BMI-1, p16/p53	–	miR-15a/16-1 down-regulation enhances BMI-1 oncoprotein up-regulation, which decreases p16 tumor suppressor and increases etoposide resistance.	–	(35)
	–	SK-N-BE(2), Shy-SY5Y, MHH-NB-11, PC3, RPMI-8266	Bcl2, cyclin D1, CCND1, ERK/CXCR4	MAPK	Up-regulation of miR-15a/16-1, regulated by CXCR4, results in the repression of BCL-2 and cyclin D1. miR-15a/16-1 increases apoptosis and reduces the proliferation and survival of tumor cells.	–	(36)
hsa-miR-34a-5p, has-let7 family, hsa-miR-16-5p, hsa-miR-20b-5p, hsa-miR-409-3p	–	SK-N-SH, LA-N-5, SK-N-BE	-/LMO1	–	These miRNAs significantly diminish cell proliferation of NB cell lines.	–	(37)
miR-146a	–	SK-N-SH, HEK293	BCL11A	–	miR-146a overexpression inhibits cell proliferation and increases the apoptosis rate of human NB cells.	–	(38)
miR-129	88 NB and 23 ANTs	NBSD, SK-N-SH, SK-SY-5Y, SK-N-AS, IMR-32, Neuro-2a, BEM17, NB1, Kelly, NB-1643, HEK293T	MYO10	–	miR-129 down-regulates MYO10 levels and then represses cell proliferation and increased chemosensitivity.	–	(39)
miR-1247	10 primary NB and the corresponding ANTs	SH-SY5Y, SK-N-SH	ZNF346	–	miR-1247 markedly decreases cell proliferation and induces cell cycle arrest and cell death.	–	(40)
miR-204	200 NB tumors	BE(2)C, SH-SY5Y, SHEP, Kelly, SK-N-AS, SK-N-FI, IMR32	MYCN	–	MYCN binds to the miR-204 promoter and represses miR-204 transcription. miR-204 directly binds MYCN mRNA and diminishes MYCN expression.	–	(41)
miR-664a-5p	–	SH-SY5Y	–	–	miR-664a-5p enhances neuronal differentiation.	–	(42)
miR-124	–	M17	β-Tubulin III, MAP2, SYN, NF-M, Nestin	–	miR-124 up-regulation increases differentiation in neuronal lineages.	–	(43)
miR-505-3p	–	N2a, U251	SRSF1	–	miR-505-3p impedes neural tumor proliferation driven by SRSF1, solely in serum-reduced condition.	–	(44)
miR-513	10 primary NB and matched ANTs	SK-N-SH, SK-N-BE2, SH-SY5Y, SK-N-AS, SK-N-DZ	GLS	–	miR-513c inhibits migration, invasion, and proliferation.	–	(45)
miR-205	28 tumor and adjacent normal tissues of NB patients	SH-SY5Y, SK-N-SH, IMR32, BE(2)-C, HUVEC	CREB1, BCL-2, MMP9	–	Expression of miR-205 is down-regulated in poorly differentiated NB tissues and those of advanced stage.	–	(46)
miR-628-3p	22 primary NB and 21 normal tissues	KCNR, HEK293T, LAN5, SH-SY5Y, SK-N-SH	MYCN	–	miR-628-3p has a tumor-suppressor characteristic and down-regulates MYCN.	–	(47)
miR-17	–	SK-N-BE(1)n, LA1-55n, KCN-83n, BE(2)-M17V, SK-N-LD, SK-N-HM, BE(2)-C, LA1-5s, SH-SY5Y, SMS-LHN, CB-JMN, SH-EP1, SMS-KCNs	N-myc/ELAVL4	–	miR-17 down-regulates N-myc mRNA and protein levels, while ELAVL4 up-regulates N-myc and is a competitive factor for miR-17.	–	(48)
miR-149	117 NB patients	SH-SY5Y, CHP-212, IMR-32, SK-N-SH, SK-N-AS, NB1691, LAN1, LAN5, LAN6	Rap1	–	Down-regulation of miR-149 expression is associated with advanced stages of primary NB tumors and poor OS.	Poor OS	(49)
miR-137	–	SH-SY5Y, SK-N-SH, MIR-32, SK-N-BE (2), normal fibroblast 3T3 cells, primary normal human astrocytes	MDR1/HDAC8	–	HDAC8 is overexpressed in NB cells and down-regulates miR-137 levels, which further decreases MDR1 and sensitivity to doxorubicin.	–	(50)
	88 NB patients	N-2a, SH-SY5Y	EZH2, CLU, NGFR	–	Resveratrol induces miR-137 up-regulation and reduces EZH2 repression. EZH2 reduction results in increased CLU ad NGFR tumor suppressors.	Lower OS	(51)
miR-143	–	SH-SY5Y	-/NO, RBM3, p38	–	RBM3 abolishes the induction of miR-143 and apoptosis.	–	(52)
miR-410	61 cases of NB and normal tissues	SK-N-BE(2), NB1691	VEGFA/SPARC	–	Concomitant SPARC up-regulation and radiation restricts tumor growth and angiogenesis by down-regulating VEGF-A *via* miR-410.	–	(53)
miR-93-5p	–	SK-N-AS	VEGF, IL-8	–	miR-93-5p is down-regulated in NB cells, which promotes VEGF and IL-8 and tumorigenesis.	–	(54)
miR-141	–	IMR-32, SH-SY5Y, S-K-NAS, NB-1691, LAN-5, LAN-6, HEK293T	FUS	–	miR-141 up-regulation inhibits cancer proliferation, cell cycle progression, tumor growth, migration, and rises cisplatin sensitivity.	–	(55)
miR-497	NRC dataset: 365 NB samples	CHLA-90, SK-N-BE(2), LA1-5s, SK-N-AS, HEK293T	WEE1, CHEK1, AKT3, BCL2, VEGFA	–	miR-497 overexpression reduces the proliferation of multiple chemoresistant NB cell lines and induced apoptosis in MYCN-amplified cell lines. Moreover, miR-497 in NB xenografts diminishes tumor growth and inhibits vascular permeabilization.	Lower progression-free survival	(56)
miR-451	37 NB and ANTs	SK−N−SH, GI−LA−N	MIF	–	miR-451 reduces cell proliferation, invasion, and migration. Reduction in miR−451 increases tumor size, dedifferentiation, lymph node metastasis, TNM stage, and remote metastases.	–	(57)
miR-203	16 NB and ANTs	SK−N−SH, SH−SY5Y	Sam68	–	Up-regulation of miR-203 inhibits the proliferation, migration, and invasion rates.	–	(58)
miR-26a-5p	200 patients with primary NB, GSE32664 dataset: 75 primary tumors	IMR5-75-shMYCN, SHEP-MYCN-ER, MYCN3, HEK293T	LIN28B/MYCN	–	MYCN overexpression reduces miR-26a-5p (not in the transcription stage), and miR-26b-5p results in LIN28B up-regulation.	Lower OS rate	(59)
miR-26b-5p							
miR-337-3p	30 primary NB cases and 21 normal dorsal ganglia	SK-N-SH, SKN-AS, SH-SY5Y, SKN-BE(2), HepG2, PC-3, HeLa, 786-O, HUVEC	MMP14, AGO2	–	miR-337-3p inhibits the activity of *MMP-14* promoter and, its nascent transcription.	Lower OS rate	(60)
miR-362-5p	12 metastatic and 12 primary NB tissues	SH-SY5Y, IMR-32, HEK293	PI3K-C2β	–	Overexpression of miR-362-5p inhibits cell proliferation, tumor growth, migration, and invasion of NB cells.	–	(61)
miR-659-3p	22 bone marrow infiltrating samples, 22 primary tumor samples	HTLA-230, SH-SY5Y	CNOT1, AKT3, BCL2, THSB2, CYR61	–	Inhibiting miR-659-3p results in over-expressed CNOT1 and down-regulated AKT3, BCL2, CYR61, and THSB2, (all involved in focal adhesion) as observed in bone marrow infiltrating NB cells.	–	(62)
miR-182-5p	100 NB patients	NGP, NGP-lv-hp53, NGP-lv-mp53, SK-N-AS, SK-N-Be(2c), IMR-32, IMR-32-lv-hp53, IMR-32-lv-mp53. NGP/IMR-lv-hp53, NGP/IMR-lv-mp53	-/p53	–	Overexpression of miR-182-5p and miR-432-5p increases apoptosis rate and promotes neuronal differentiation.	–	(63)
miR-432-5p						Lower progression-free survival	
miR-449a	Versteeg cohort: 88NB patients, Kocak cohort: 476 NB patients	BE(2)-C, SKNBE and BE(2)-M17, LAN6, KELLY	MFAP4, PKP4, TSEN15, CDK6, LEF1	–	miR-449a impedes NB cell survival and proliferation by increasing cell differentiation and cell cycle arrest.	–	(64)
miR-520f	GSE16476 dataset: 237 NB patients, 3 FFPE matched pre-treatment and post-treatment	SK-N-AS	NAIP	–	miR-520f down-regulation increases NAIP levels. miR-520f levels are determined to be significantly lower in post-chemotherapy treatment.	–	(65)
miR-542-3p	69 primary NB tumors	IMR-32, SHEP, SK-N-BE and WAC II, HEK293, SK-N-SH, SH-SY5Y	Survivin	–	Up-regulation of miR-542-3p in NB cells diminishes the cell viability and proliferation, induced apoptosis, and down-regulates Survivin.	Lower survival rate	(66)

Schulte et al. have identified seven miRNAs whose expressions have been increased by MYCN *in vitro* and are over-expressed in primary neuroblastomas that harbor *MYCN* amplification. Notably, three of them were from the miR-106a and miR-17 clusters whose expressions are controlled by c-Myc. They also demonstrated up-regulation of miR-221 by MYCN in neuroblastoma (67). Montana et al. have shown transactivation of the miRNA 17-5p-92 cluster by MYCN. These miRNAs have further been demonstrated to suppress expression of p21 and BIM, thus influencing cell cycle transition and apoptosis, respectively. Notably, forced up-regulation of miRNA 17-5p-92 cluster in neuroblastoma cell lines that do not harbor *MYCN* amplification enhances their tumorigenic potential in animal models. On the other hand, suppression of miR-17-5p attenuates the proliferation of *MYCN*-amplified neuroblastoma cells *via* up-regulation of p21 and BIM. Over-expression of miR-17-5p has also been verified in primary neuroblastoma patients especially those with *MYCN* amplification and poor clinical outcome (68). miR‐640, miR‐543, miR‐624‐3p, and miR‐196‐b are among up-regulated miRNAs in neuroblastoma. Notably, these miRNAs target ING5 transcript. miRNA‐ING5‐histone acetylation axis has been recognized as the main route through which two anti-cancer drugs namely a histone deacetylase inhibitor and a proteasome inhibitor block progression of neuroblastoma (69). miR-1303 is another over-expressed miRNA in neuroblastoma. Up-regulation of this miRNA enhanced proliferation of neuroblastoma cells through targeting GSK3β and SFRP1. miR-1303 also increased levels of MYC and CyclinD1, and diminished p21 and p27 levels (70). **Table 2** lists up-regulated miRNAs in neuroblastoma.

**Table 2:** Up-regulated miRNAs in neuroblastoma (NB, neuroblastoma; OS, overall survival).

miRNA	Number of clinical samples	Assessed cell line	Targets/regulators	Signaling pathway	Function	Effect of miRNA up-regulation on patients’ prognosis	Ref
miR-25	Versteeg dataset: 88 samples, Kocak dataset: 649 samples, SEQC dataset: 498 samples	SH-SY5Y	Gsk3β/SLC34A2	Wnt	SLC34A2 inhibits the stemness of NB cells *via* the miR-25–Gsk3β axis.	–	(71)
miR-640, miR‐543, miR‐624‐3p, miR‐196‐b	50 NB tissues	SH-SY5Y, SK‐N‐AS, NGP, SK‐N‐BE2	ING5	–	Suberoylanilide hydroxamic acid downregulates these miRNAs to induce ING5 overexpression.	–	(69)
miR-3613−3p	–	BE(2)-C, Kelly, IMR−32, SK−N−SH, CHP−134, LAN−1, LAN−5, PC3	APAF1, DICER, DFFB, VHL, NF1/MCPIP1	Wnt, TGFβ, Akt	The up-regulation of miR-3613-3p increases viability but reduces the apoptosis of NB cells.	–	(72)
miR-181a/b	32 primary NB tissues and 6 gangliocytoma tissues as controls	SK-SY5Y, SK-N-SH, BE(2) C, IMR-32, HUVEC, HEK293T	ABI1	–	High miR-181a/b expression markedly enhances the proliferation, tumorigenesis, progression, migration, and invasion of NB cells, though it reduces the apoptosis rate. MYCN amplification and miR-181a expression are correlated.	–	(73)
	–	SH-SY5Y	p38MAPK/triptolide	NF-κB	Through down-regulating miR-181a/b level, Triptolide inhibits cell viability, proliferation, and migration, but induces cell apoptosis.	–	(74)
miR-181a	–	SH-SY5Y, A172, U251	PARK2	–	miR-181a suppresses mitochondrial uncoupling agents-induced mitophagy by decreasing the destruction of mitochondrial proteins.	–	(75)
miR-221	31 NB tissues	SK-N-AS, SK-N-DZ, IMR-32, HEK293T, SH-SY5Y	LEF1, NLK, p21, p27, p57	Wnt	miR-221 diminishes LEF1 phosphorylation but up-regulates MYCN. Overexpression of miR-221 enhances the cell cycle transition especially in S-phase, promoting the proliferation of NB cells.	Poor survival rate	(76)
miR-558	30 primary NB and 10 ganglioneuroblastoma samples, GSE62564 database: 498 NB cases	NB-1643, SK-N-BE(2), NB-1691, IMR32, BE(2)-C, SK-N-AS, SH-SY5Y, SK-N-SH, HUVEC	AGO2, HIF-2α	–	miR-558 enhances the proliferation, invasion, metastatic capacities, and angiogenic potential.	Poor OS	(77)
	30 primary NB cases	SK-N-SH, SK-N-AS, SH-SY5Y, SK-N-BE(2), HUVEC	HPSE, VEGF, AGO1	–	Knock-down of endogenous miR-558 reduced the proliferation, invasion, metastasis, and angiogenic potential.	–	(78)
miR-1303	8 NB and adjacent normal nerve tissues	U343, SK-N-SH, SH-SY5Y, LAN5, IMR-32, SH-EP	GSK3β, SFRP1, p21, p27, MYC, CyclinD1	–	miR-1303 overexpression results in up-regulated proliferation rates.	–	(70)
miR-19b	–	SH-SY5Y, BE(2)-M17	p-AKT, PTEN	mTOR	AZD8055 significantly reduces miR-19b and p-AKT expression and enhances the cytotoxic activity of mTOR inhibitors and PTEN levels. miR-19b overexpression reverses mTOR inhibitors toxicity and cell viability.	–	(79)
miR-21			CHL1		miR-21 promotes the proliferation and invasion of NB cells.		(80)

Aberrant expression of miRNAs in neuroblastoma samples can be used as biomarkers for prediction of the course of malignancy. For instance, down-regulation of miR-490-5p has been correlated with INSS stage, lymph node involvement, and poor clinical outcome of patients with neuroblastoma (22). Similarly, decreased expression of miR-186, let-7, miR-497 and miR-432-5p predicts lower survival rates (31, 34, 56, 63). **Table 3** reviews the results of studies which evaluated this aspect of miRNAs.

**Table 3:** Diagnostic importance of miRNAs in neuroblastoma (NB, neuroblastoma; OS, overall survival; EFS, event-free survival).

Sample number	Kaplan–Meier analysis	Reference
miR-490-5p expression in NB patients: 21 high and 51 low	Higher miR-490-5p expression levels markedly correlate with higher survival rate.	(22)
miR-323a-5p expression: high in 228 and low in 25 NB patients	Higher expression levels of miR-323a-5p expression correlates with higher OS rate.	(27)
miR-2110 expression in NB patients, derived from SEQC dataset: high=406, low=92	Higher expression levels of miR-2110 correlate with lower OS.	(30)
miR-186 expression in NB patients: 235 high and 263 for EFS, 298 high and 200 low for OS	Low levels of miR-186 correlate with poor OS and EFS.	(31)
miR-149 expression in NB patients: low=59, high=58	Higher miR-149 expression level significantly correlates with higher OS rate.	(49)
miR-221 expression in NB patients: low=17, high=14	miR-221 expression level negatively correlates with survival ratio.	(76)
miR-181c expression: high=326, low=172	Higher miR-181c significantly correlates with higher OS in NB patients.	(81)
miR-558 expression in two sets of samples: 13 low and 17 high, 170 low and 328 high	Higher expression negatively correlates with the OS rate.	(77)
Let-7 expression in NB patients: normal levels=60, loss of Let-7 = 112	Loss of Let-7 expression correlates with lower OS rate.	(34)
70 patients with NB, divided into 3 groups based on their expression level of miR-21 and risk: low=22, moderate=23, high=25	In patients with NB, higher miR-21 expression correlated with lower rates of OS.	(82)
miR-497 expression in NB patients from NRC dataset: high=100, low=228	Lower miR-497 expression correlates with lower progression-free survival rates.	(56)
miR-26a-5p expression in NB patients: high=44, low=48	Lower miR-26a-5p and miR-26b-5p expression correlate with lower OS rate.	(59)
miR-26b-5p expression in NB patients: high=50, low=42		
miR-337-3p expression in 30 NB patients: 17 high and 13 low	Lower miR-337-3p levels correlate with lower OS rate.	(60)
miR-432-5p expression in 100 NB patients	Lower miR-432-5p expression levels relates to lower cumulative survival.	(63)
miR-137 expression in NB patients: 17 high and 71 low	miR-137 expression negatively correlates with OS rate.	(51)
miR-542-3p expression level in NB patients: 34 high and 34 low	miR-542-3p over-expression significantly correlates with better survival rate.	(66)
miR-34a expression in NB patients: 15 high and 15 low	Higher miR-34a level significantly correlates with better survival rate.	(18)

### Dysregulated lncRNAs in Neuroblastoma

LncRNAs can regulate expression of genes *via* different mechanisms including alterations in chromatin configuration, modulation of transcription, splicing, mRNA stability and bioavailability as well as post-translational modifications (83). Therefore, they contribute in the pathogenesis of human cancers. Prajapati et al. have analyzed RNA-seq data of a number of neuroblastoma samples to recognize their differential expression in among primary neuroblastoma, relapsed ones and metastasized tumors. They reported up-regulation of RFPL1S, PPP1R26-AS1, RP11-439E19.3, CASC15, AC004540.5, and CTD-2881E23.2 while down-regulation of USP3-AS1, CHRM3-AS2 and RP6-99M1.2 in tumor cells compared with the corresponding non-tumor mononuclear cells isolated from bone marrow (MNCs). Moreover, expression of theses up-regulated lncRNAs along with ZRANB2-AS2 and LINC00511 were increased in the disseminated tumor cells (DTCs) compared with the corresponding MNCs. They suggested CASC15, PPP1R26-AS1, and USP3-AS1 lncRNAs as putative markers in clinical investigations in this type of pediatric cancer (84). Pandey et al. have assessed transcript signature of low-risk and high-risk neuroblastoma samples. They have reported association between a certain lncRNA namely neuroblastoma associated transcript-1 (NBAT-1) and prognosis of neuroblastoma. Altered expression of this lncRNA between the mentioned groups of neuroblastoma has been attributed to CpG methylation and the presence of a certain functional polymorphism on chr 6p22. Mechanistically, NBAT-1 down-regulation enhances proliferation and invasion of neuroblastoma cells through suppression of expression of target genes as well as induction of expression of neuronal-specific transcription factor NRSF/REST (85). Liu et al. have reported co-amplification of the lncUSMycN with MYCN in a portion of human neuroblastoma samples. This lncRNA has been shown to bind with the RNA-binding protein NonO, resulting in N-Myc up-regulation (86). Barnhill et al. have revealed that low levels of CAI2 expression in normal tissues in spite of its over-expression in the majority of tumor cell lines with a normal 9p21 locus. This lncRNA has been suggested to modulate expression of p16 and/or ARF. CAI2 expression has been higher in advanced-stage neuroblastomas in an independent manner from *MYCN* amplification (87). Watters et al. have shown modulation of expression of several transcribed Ultra-conserved regions (T-UCRs) in response to all-trans-retinoic acid (ATRA). Among these transcripts has been the lncRNA T-UC.300A which has imperative impacts in the regulation of cell proliferation, invasion and the suppression of differentiation of neuroblastoma cells before exposure to ATRA (88). Yu et al. have identified a transcript which has been over-expressed in neuroblastoma and named it the non-coding RNA expressed in aggressive neuroblastoma (ncRAN). Over-expression of this transcript has been associated with poor survival of patients. This lncRNA has been mapped to the region of 17q which is amplified in neuroblastoma and exerts oncogenic effects in this type of cancer (89). **Tables 4** and **5** enlist over-expression and decreased expression lncRNAs in neuroblastoma, respectively.

**Table 4:** Up-regulated lncRNAs in neuroblastoma (ANT, adjacent normal tissue; NB, Neuroblastoma; EMT, epithelial-mesenchymal transition; OS, overall survival; EFS, event-free survival).

lncRNA	Specimens	Cell lines	Targets/regulators	Signaling pathway	Function	Effect of lncRNA up-regulation on patient’s prognosis	Ref
DLX6AS1	70 pairs of primary NB and ANTs	SK-N-SH, SH-SY5Y, SK-N-AS, SK-N-BE, HEK293T	miR-497-5p, YAP1	–	DLX6-AS1 knock-down results in diminished proliferation rate, tumor proliferation, migration, EMT, and invasion.	Poor prognosis and OS	(90)
	31 NB and ANTs	SK-N-SH, LAN-6, HUVEC	miR-506-3p, STAT2, CDK1, Cyclin D1	–	DLX6-AS1 silencing inhibits proliferation, tumor growth, cell cycle, and glycolysis.	–	(91)
lncNB1	SEQC-RPM-seqcnb1 dataset: 493 NB tissues	BE(2)-C, IMR32, SY5Y, SHEP, HEK293T	RPL35, E2F1, DEPDC1B, ERK, n-Myc	–	LncNB1 down-regulation abrogates clonogenic capacity and leads to NB tumor regression.	Lower OS	(92)
DEIN	Case study of a monozygotic twin with NB	–	HAND2	–	Both twin liver tumors had a 4q34.1 amplification of DEIN, which is strongly linked to HAND2. HAND2 functions as an essential regulator of neurogenesis.	–	(93)
LINC01296	28 patients with primary NB, R2: Genomics Analysis and Visualization Platform for 88 NB patients	–	–	–	Over-expression of LINC01296 was associated with age>18 month and advanced INSS stage. Moreover, LINC01296 over-expression is correlated with larger tumor size, elevated serum lactate dehydrogenase level, and serum neuron-specific enolase level.	Poor prognosis and OS	(94)
SNHG16	40 patients with NB, GSE62564 dataset: 498 NB patients	SH-SY5Y	–	–	SNHG16 down-regulation inhibits proliferation, migration, and induces cell cycle arrest at the G0/G1 phase. SNHG16-related RNA binding proteins partake in controlling mRNA metabolic processes, gene silencing, mRNA transport, RNA splicing, and translation.	Poor OS and EFS	(95)
	76 NB tissues	SK-N-AS, SK-N-SH, SK-N-AS-R, SK-NSH-R	miR-338-3p, PLK4, MRP1, p-glycoprotein	PI3K/AKT	In cisplatin-resistant NB tissues and cells. SNHG16 is up-regulated, while miR-338-3p is down-regulated.	–	(96)
	48 NB and 38 ANTs	SK-N-SH, IMR‐32, SK-N-AS, SK-N-DZ, HUVEC	HOXA7, miR-128-3p	–	SNHG16 silencing represses proliferation, migration, and invasion but boosts apoptosis.	–	(97)
	30 NB and 30 ANTs	SKNBE-2, SK-N-SH, HEK293, LAN-5	miR-542-3p, HNF4α	RAS/RAF/MEK/ERK	The Knock-down of SNHG16 or HNF4α impedes proliferation, migration, invasion, and EMT.	–	(98)
	45 NB and ANTs	LAN-1, SHEP, SKN-SH, IMR-32, HUVEC	miR-542-3p, ATG5	–	The knock-down of SNHG16 diminishes proliferation, migration, invasion, autophagy, and tumor growth.	Lower OS	(99)
MIAT	–	Neuro2A	caspase-3, miR-211, GDNF	–	MIAT overexpression lowers the apoptosis rate.	–	(100)
SNHG7			miR-653-5p, STAT2		SNHG7-miR‐653‐5p‐STAT2 loop is involved in regulation of NB progression.		(101)
	26 NB and ANTs	SK-N-AS, LAN-6, HUVEC	miR-329-3p, MYO10	–	Silencing of SNHG7 reduced cisplatin resistance and suppressed cisplatin-induced autophagy.	–	(102)
	45 NB and ANTs	SH-SY5Y, SK-N-SH, NB-1, SK-N-AS, HUVEC	miR-323a-5p, miR-342-5p, CCND1	–	SNHG7 knock down repressed migration, invasion, and glycolysis.	Poor prognosis and OS	(103)
RMRP	44 cases of neonatal NB and ANTs	NB-1, SK-N-AS, HEK293T	miR-206, TACR1	ERK1/2	RMRP knock-down lessens proliferation, migration, and invasion rates. RMRP expression is markedly increased in patients with advanced neonatal NB versus early stages.	Poor OS	(104)
SNHG1	–	SK-N-DZ, SK-N-BE(2)C, SK-N-AS	MATR3, YBX1, HNRNPL	–	SNHG1 significantly elevates ribonucleoprotein complex biogenesis, RNA processing, and RNA splicing.	–	(105)
	GSE62564 dataset: 493 NB patients, GSE12460 dataset: 47 NB patients	SK-N-DZ, SK-N-SH, SK-N-BE(2)-C, SK-N-AS, SK-N-F1	-/MYCN	–	MYCN amplification up-regulates SNHG1.	Poor OS and EFS	(106)
GALNT8	TCGA dataset: 88 NB cases	SK-N-AS, HEK293T	TCEA1, RBMX, MCM2, CBX3	–	Suppressing the GAU1/GALNT8 cluster hinders tumor progression and growth. GAU1 recruits TCEA1 to activate GALNT8 expression.	Poor OS	(107)
GAU1							
MYCNOS-01	88 NB samples	KELLY, SY5Y	MYCN	–	MYCNOS-01 suppresses MYCN protein levels. The suppression of MYCNOS-01 or MYCN expression reduced cell proliferation and viability.	–	(108)
pancEts-1	42 NB patients and 88 NB cases from GSE16476 dataset	NB-1643, SK-N-BE(2), NB-1691, IMR32, BE(2)-C, (SK-N-AS, SH-SY5Y, SK-N-SH	hnRNPK, β-catenin	–	PancEts-1 increases the proliferation, invasion, and metastasis of NB cells. pancEts-1 binds to hnRNPK to enhances its interplay with β-catenin and stabilizes the β-catenin.	Poor survival	(109)
MALAT1	15 normal tissues, 19 primary NB, and 28 metastatic NB tissues	NGP, SH-SY5Y, NMB, SHEP21N, SKNAS, SHEP2, HEK293T	Axl, AKT, ERK1/2	–	MALAT1 overexpression increases invasion and migration.	–	(110)
	–	BE(2)-C, HUVEC	FGF2	–	MALAT1 significantly promotes cell migration, invasion, and vasculogenesis.	–	(111)
	–	BE(2)-C, CHP134	-/N-Myc, JMJD1A	–	Migration and invasion rate increase following MALAT1 overexpression.	–	(112)
GAS5	–	IMR-32, CHLA-122, SMS-KAN, SK-N-Be(1), KCNA, NPE, SK-N-AS, LA-N-6, CHLA-15, SK-N-FI, CHLA-171, NB-EBc1, CHLA-42, GI-M-EN	p53, BRCA1, GADD45A, HDM2	–	GAS5 loss results in defects in cell proliferation, apoptosis, but induces cell cycle arrest.	–	(113)
HCN3	Tumor and para-tumor tissue samples (n = 6)	BE(2)-C	BID, Noxa, HIF-1α	–	Linc01105 knock-down increases HIF-1α and promotes cell proliferation. In contrast, linc01105 and HCN3 knock-down increase the apoptosis rate.	–	(114)
linc01105							
lncUSMycN	Versteeg dataset: 88 NB samples, Kocak dataset: 476 NB samples	BE(2)-C	NCYM, N-myc, NonO	–	LncUSMycN up-regulates NCYM expression.	–	(115)
	47 primary NB samples, Versteeg dataset: 88 NB tissues, Kocak dataset: 476 NB tissues	IMR32, BE2C, SK-N-DZ, CHP134, Kelly, SK-N-FI, SK-N-AS, NB69, SY5Y, SHEP, LAN-1	NonO, N-Myc	–	lncUSMycN increase up-regulates N-Myc RNA and NB cell proliferation.	Poor OS	(86)
HOXD-AS1	GSE3446 dataset: 102 NB patients	SH-SY5Y	MAGEA9B, SNN, TMEM86A, VIPR1, CREM, TSPAN2, CNR1, CREBL1, PTGS1, ADAMTS3, AMDMD2, ANG, ASNA1/retinoic acid	PI3K/Akt, JAK/STAT	Following RA treatment, HOXD-AS1 diminishes the expression of genes involved in NB progression, angiogenesis, and inflammation.	–	(116)
CAI2	62 primary NB samples and 25 healthy controls	FS15, NMB7	P16, ARF	–	CAI2 expression is significantly higher in advanced-stage NB.	Poor OS	(87)
Paupar	–	N2A	KAP1, PAX6, RCOR3, PPAN, CHE-1, ERH	–	Paupar regulates expression of some target genes involved in the regulation of neuronal function and cell cycle.	–	(117)
	–	N2A	PAX6, E2f2, E2f7, Cdc6, Cdkn2c, Kdm7a, Sox1, Sox2, Hoxa1, Hes1	–	Paupar silencing disrupts the cell cycle transition and stimulates neuron differentiation.	–	(118)
NORAD	38 pairs of NB and normal tissues	SK-N-SH, IMR-32, HUVEC	MiR-144-3p, HDAC8	–	NORAD enhances the proliferation, tumor growth, metastasis, and doxorubicin resistance, though it restricts apoptosis and autophagy.	–	(119)
CASC11	42 neonatal NB and 42 normal tissues	SK-N-AS and NB-1, hTERT-RPE1	miR-676-3p, NOL4L, AGO2	–	CASC11 depletion represses cell proliferation and invasiveness.	Poor survival	(120)
DUXAP8	45 NB patients, at 1 + 2+4S stage (n = 18) and 3 + 4 stage (n = 27)	SK-N-SH, IMR-32, HUVEC, HEK293T	miR-29, NOL4L	Wnt/β-catenin	DUXAP8 expression is positively related to the stage of NB tumors and is negatively associated with the survival rate of NB patients. DUXAP8 knock-down reduces the proliferation, colony formation, cycle, and motility of NB cells.	Lower OS	(121)
SNHG4	30 primary NB and ANTs	SH-SY5Y, CHP-212, SK-N-FI, IMR-32, HEK293T	miR-377-3p	–	LncRNA SNHG4 escalates NB proliferation, migration, EMT, and invasion and reduces the apoptosis rate.	Lower survival rate	(122)
lncNB	476 NB patients		BMX		The super-enhancer driven long non-coding RNA lncNB promotes neuroblastoma tumorigenesis.	Poor prognosis	
NHEG1	GSE62564 dataset: 498 patients, 42 primary NB cases and 21 normal dorsal ganglia	MCF-10A, SK-N-BE(2), IMR32, BE(2)-C, NB-1643, NB-1691, SH-SY5Y, SK-N-SH, SK-N-AS, HCT116	DDX5, β-catenin/LEF1, TCF7L2	Wnt/β-catenin	NHEG1 depletion accelerates differentiation and inhibits the proliferation and aggressiveness of NB cells.	Lower OS and EFS	(123)
XIST	30 NB and ANTs	SK-N-BE(2), HEK293, GI-LI-N	HK2, miR-653-5p	–	XIST knock-down curtails tumorigenesis by suppressing proliferation and invasion. It also increases the radiosensitivity by diminishing colony constuction and glycolysis.	–	(124)

**Table 5:** Down-regulated lncRNAs in neuroblastoma (NB, Neuroblastoma; OS, overall survival; EFS, event-free survival).

lncRNA	Specimens	Cell line	Targets/regulators	Signaling pathway	Function	Effect of lncRNA down-regulation on patient’s prognosis	Reference
NR_120420	–	SH-SY5Y	P65, ERK, AKT	NF-κB	The knock-down of NR_120420 enhances cell viability but reduces the apoptosis.	–	(125)
CASC15	220 high-risk NB samples	SK-N-BE2, SK-N-SH	NEUROD1, NEDD9, NEUROG2	–	CASC15 depletion improves proliferation and invasive capabilities and shifts the NB gene expression away from the differentiated neural phenotype.	Lower OS	(126)
	Two cohorts: one with 59 and the other with 498 NB patients	SHSY-5Y, SK-N-AS, IMR32, SK-N-BE2, hESCs, HEK293T	SOX9, CHD7, USP36	–	These lncRNAs regulate SOX9 expression through regulation of CHD7 stability. Loss of this synergy between these lncRNAs enhances proliferation, migration, invasion, colony formation of NB cells.	Poor OS and EFS	(127)
NBAT1							
FOXD3-AS1	42 NB tumor samples, GSE16476 dataset: 88 cases of NB	NB-1643, SK-N-BE(2), NB-1691, IMR32, BE(2)-C, SK-N-AS, SH-SY5Y, SK-N-SH	PARP1, CTCF	–	Over-expression of FOXD3-AS1 promotes neuronal differentiation and reduces aggressive behavior of these cells.	Poor survival	(128)
MEG3	Tumor and para-tumor tissue samples (n = 6)	BE(2)-C	PMAIP1, BID, HIF-1α	–	MEG3 overexpression reduces proliferation and elevates apoptosis rate.	–	(114)
Linc-NeD125	–	BE(2)-C, D283Med, NB4, HL-60	BCL-2	–	Linc-NeD125 is the host gene of miR-125b-1. Its down-regulation reduces cell proliferation and activates the antiapoptotic factor BCL-2.	–	(129)
MYCNOS	–	Lan6	MYCN, MAP4, G3BP1, FKBP3	–	MYCNOS RNA localizes to the MYCN promoter and reduces its expression.	–	(130)
CASC15-S	NCI TARGET project: 108 NB patients	SK-N-BE2, SK-N-SH, HEK293T	ALCAM, NEUROD1, NEDD9, NEUROG2	–	Attenuating CASC15-S elevates cellular proliferation, proliferation, invasion, and migratory capacity. CASC15-S regulates genes involved in neural crest development.	Poor OS	(126)
NBAT-1	15 NB snap-frozen tumors, 108 patients and RNA-seq data of 498 patients	SK-N-FI, SH-SY5Y, SK-N-AS, SK-N-BE(2)	NRSF, REST, SOX9, VCAN, EZH2	–	NBAT-1 down-regulation boosts cellular proliferation and invasion and inhibits neuronal differentiation.	Poor survival	(85)
CASC7	48 NB patients	LAN-2	miR-10a, PTEN	–	CASC7 overexpression decreases the proliferation of NB cells.	–	(131)
KCNQ1OT1	Xena datahub: 128 NB tissues	SH-SY5Y, IMR32, HEK293T	miR-296-5p, Bax	–	KCNQ1OT1 acts as a sponge for miR-296-5p. miR-296-5p inhibits Bax protein and cell apoptosis.	–	(132)
NEAT1	30 NB tissues	SKN-SH, SH-SY5Y, IMR-32, SH-N-AS	miR-183-5p, FOXP1	ERK/AKT	NEAT1 up-regulation lowers cell proliferation, migration, and invasion rates.	–	(133)

Dysregulation of several lncRNAs in neuroblastoma samples has been correlated with survival of patients. For instance, high levels of DLX6-AS1, lncNB1, LINC01296, SNHG16 and RMRP expression have been linked with poor prognosis and lower survival (90, 92, 94, 95, 104). **Table 6** summarizes the results of studies which assessed correlation between expression levels of lncRNAs and survival of patients with neuroblastoma.

**Table 6:** Prognostic value of lncRNAs in neuroblastoma (NB, neuroblastoma; OS, overall survival; EFS, event-free survival).

Sample number	Kaplan–Meier analysis	Multivariate cox regression	Reference
Two groups of 35 patients, each expressing low and high levels of DLX6AS1	High DLX6-AS1 expression correlates with a low OS rate of NB patients.	–	(90)
EQC-RPM-seqcnb1 dataset: 246 low and 247 high expression groups of lncNB1	High levels of lncNB1 correlate with poor prognosis.	–	(92)
Genomics Analysis and Visualization Platform for NB dataset: Two groups of high (=21) and low (=67) for LINC01296 expression	High LINC01296 expression is associated with poor outcome.	–	(94)
GSE62564 dataset: two groups of low and high expression for SNHG16 expression, each containing 249 patients	High levels of SNHG16 expression correlates with lower EFS and OS.	–	(95)
High (=34) and low (=10) expressing groups of RMRP lncRNA	Higher RMRP expression relates to poor prognosis and survival.	–	(104)
GAU1 expression in two groups from TCGA dataset: high (=44) and low (=44)	High GAU1 expression correlates with lower OS.	–	(107)
GALNT8 expression in two groups from TCGA dataset: high (=13) and low (=75)	Higher GALNT8 levels correlate with poor OS.		
CASC15 expression: Cohort a: high (=29) and low (=30) expression groups Cohort b: high and low groups, each containing 249 patients	Lower levels of CASC15 expression correlate with lower OS and EFS.	Both CASC15-003 and CASC15-004 predict OS and EFS.	(127)
GSE16476 dataset: 88 NB cases, expressing high (=22) and low (=66) levels of FOXD3-AS1 42 NB patients expressing high (=19) and low (23) levels of FOXD3-AS1	Lower expression levels of FOXD3-AS1 correlate with lower OS.	FOXD3-AS1 is a possible independent prognostic factor.	(128)
pancEts-1 expression: 42 NB patients (low=23, high=19) and 88 NB cases (low=50, high=38) from GSE16476 dataset	Higher levels of pancEts-1 negatively correlate with survival rate.	Patients’ age, MYCN amplification, INSS stage, pancEts-1 expression, and hnRNPK expression, but not gender, are independent prognostic factors for poor outcome.	(109)
SNHG1 expression in GSE62564 dataset: 246 low and 247 high, GSE16476 dataset: 44 low and 44 high	Higher expression levels of SNHG1 negatively correlate with OS and EFS.	SNHG1 high expression is a significant low hazard rate indicator for both OS and EFS.	(106)
CAI2 expression in NB patients: high=19, low=43	CAI2 expression negatively correlates with OS and EFS.	–	(87)
NBAT-1 expression in 2 cohorts: 1) 50 high and 43 low, 2) 314 high and 184 low	NBAT expression significantly correlates with OS and EFS.	NBAT-1 is an independent prognostic marker in predicting EFS.	(85)
lncUSMycN expression: Versteeg dataset: 79 low and 9 high, Kocak dataset: 429 low and 47 high	High levels of lncUSMycN expression have been linked with poor survival.	High levels of lncUSMycN and NonO expression in are linked with poor OS, independent of disease stage, age at diagnosis, and MYCN amplification.	(86)
CASC15-S expression in NB patients: low=163, high 87	Higher levels of CASC15-S significantly correlate with longer OS in NB patients.	–	(126)
CASC15-S expression: 163 low, 87 high	CASC15-S expression in NB patients significantly correlates with OS.	CASC15-S expression is correlated with more aggressive features and lower OS.	(126)
CASC11 expression in NB patients: 21 high and 21 low	CASC11 expression negatively correlates with the survival rate.	–	(120)
DUXAP8 expression in two groups: group 1: 1 + 2+4S stage (n = 18) and group 2: 3 + 4 stage (n = 27)	The survival rate is low in high expression of the DUXAP8 group compared with lower expression of the DUXAP8 group.	–	(121)
SNHG7 expression level in NB patients: 25 high and 20 low	SNHG7 expression levels negatively correlate with the OS rate.	–	(103)
NHEG1 expression: GSE62564 dataset: 498 patients (432=low, 66=high), 42 primary NB cases and 21 normal dorsal ganglia	NHEG1 expression negatively correlates with OS and EFS rates.	NHEG1 expression has a significant prognostic value for NB patients.	(123)
SNHG16 expression in NB patients: high=22, low=23	SNHG expression levels negatively correlates with OS.	–	(99)

### Expression and Function of circRNAs in Neuroblastoma

Circular RNAs (circRNAs) constitute a group of ncRNAs which are produced from exons or introns through construction of covalently-closed circles (134). Recent studies have shown dysregulation of this type of ncRNAs in cancers. For instance, circDGKB has been shown to be over-expressed in neuroblastoma tissues versus normal dorsal root ganglia. Notably, over-expression of this circRNA has been an indicator of poor survival of these patients. Mechanistically, circDGKB enhances cell proliferation, migration and invasion of neuroblastoma cells while inhibiting cell apoptosis. Moreover, up-regulation of circDGKB reduced expression level of miR-873 and increased GLI1 expression (135). **Table 7** recapitulates the results of studies which assessed function of circRNAs in neuroblastoma.

**Table 7:** List of circRNAs dysregulated in neuroblastoma.

circRNA	Pattern of expression	Samples	Cell line	Targets/regulators	Function	Patient’s prognosis	Reference
circDGKB	↑	30 NB tissues and 10 normal dorsal root ganglia as controls	SK-N-SH, SH-SY5Y	miR-873, GLI1, ZEB1	circDGKB up-regulation improves the proliferation, migration, invasion, and tumorigenesis, though it reduces cell apoptosis.	Lower OS	(135)
circ-CUX1	↑	54 NB patients, GSE16476 dataset: 88 NB patients, oncogenomic database: 117 NB and 3 normal tissues	MCF 10A, HeLa, SH-SY5Y, IMR32, SK-N-AS, BE(2)-C, SK-NMC, LoVo, PC-3, HEK293, HEK293T	EWSR1, MAZ, CUX1	circ-CUX1 knock-down inhibits aerobic glycolysis, proliferation, progression, and aggressiveness of NB. circ-CUX1 binds to EWSR1 to enable its contact with MAZ, leading to transactivation of MAZ and transcriptional modification of CUX1 and other genes linked with cancer progression.	Lower survival rate	(136)

### Polymorphisms Within ncRNAs and Risk of Neuroblastoma

Single nucleotide polymorphisms (SNPs) within lncRNAs or miRNAs can modulate expression or activity of these transcripts, thus being implicated in the development of neuroblastoma. The role of a number of SNPs within lncRNAs such as LINC00673, H19, MEG3 and HOTAIR has been evaluated in this regard (137–140). Moreover, the rs4938723 within miR-34b/c has been associated with risk of this kind of cancer (141). Notably, some studies have appraised these associations in certain subgroups of patients. For instance, the association between rs4938723 TC and CC genotypes is prominent in all age-based subgroups, both sexes, retroperitoneal tumors as well as tumors originated from other sites, and all clinical stages (141). Such detailed analyses have not been done for all assessed SNPs. **Table 8** summarizes the results of studies which assessed contribution of SNPs within ncRNAs in conferring the risk of neuroblastoma.

**Table 8:** Polymorphisms within non-coding RNAs and risk of neuroblastoma.

lncRNA/miRNA	Number of clinical samples	SNP ID	Nucleotide change	OR (95%CI)	p-value	Description	Reference
LINC00673	700 cases and 1516 controls	rs11655237	C>T	1.58 (1.06–2.35)	0.024	Patients with the T allele are considerably more prone to develop NB. A substantial association exists between rs11655237 CT/TT and NB risk in subgroups of males, adrenal gland tumors, and patients with stage IV disease.	(137)
H19	393 NB patients and 812 healthy controls	rs2839698	G>A	–	–	Separated and combined analyses indicated no associations between these polymorphisms and NB susceptibility. Only female children with rs3024270 GG genotypes had a raised NB risk.	(138)
		rs3024270	C>G	1.61 (1.04-2.50)	0.032		
		rs217727	G>A	–	–		
MEG3	392 NB children and 783 controls	rs7158663	G>A	–	–	Patients with rs4081134 AG/AA genotypes were significantly prone to develop NB among subgroups with age >18 months and stage III+IV. Carriers of these two polymorphisms were more prone to NB. These associations were found in children more than 18 months and with clinical stages of III+IV.	(142)
		rs4081134	G>A	NB developments: 1.36 (1.01-1.84), clinical stage III+IV: 1.47 (1.08-1.99)	0.042 and 0.014 respectively		
CAC15-S	250 primary NB, 20 NB cell lines	rs9295534	T>A	1.63 (1.4-1.89)	3.51×10^-12^	This polymorphism is located upstream of CASC15-S and spans regulatory chromatin and dense transcription factor binding site. This genomic area has an enhancer-like activity that is disturbed by NB risk allele.	(126)
HOTAIR	393 NB and 812 healthy controls	rs12826786	C>T	1.98 (1.14-3.42)	0.015	These polymorphisms are markedly associated with increased NB risk. In stratification analyses, these associations are more dominant in females and among patients with tumors in the retroperitoneal or mediastinal tumors.	(140)
		rs874945	C>T	1.91 (1.10-3.32)	0.022		
		rs1899663	C>A	1.87 (1.05-3.32)	0.033		
LINC00673	393 NB and 812 healthy controls	rs11655237	C>T	NB risk: 1.51 (1.06-2.14), stage IV disease: 1.60 (1.12-2.30)	0.021 and 0.011 respectively	Carriers of rs11655237 T allele are prone to NB. Associations were found in patients with adrenal gland tumors and stage IV disease.	(143)
uc003opf.1	275 patients and 531 controls	rs11752942	A>G	0.74 (055-0.99)	0.045	rs11752942 G allele is negatively related to NB risk and is more prominent in females, subjects with tumors in the mediastinum or early-stage. Besides, rs11752942 G is associated with deceased levels of LRFN2 transcripts.	(144)
CASC15 and NBAT1	36 NB patients and NB cell lines	rs6939340	A>G	–	–	This polymorphism results in lowered expression of CASC15 and NBAT1.	(127)
NBAT1	51 high-risk primary tumors and NB cell lines	rs6939340	A>G	–	P < 0.05	Lowered NBAT-1 expression in high-risk tumors relates to rs6939340.	(85)
Lnc-LAMC2–1:1	393 NB and 812 healthy cases	rs2147578	C>G	1.33 (1.01-1.75)	0.045	rs2147578 rises NB susceptibility. Children under 18 months and females have increased NB risk.	(145)
miR-34b/c	162 NB and 270 healthy controls	rs4938723	T>C	0.49 (0.33-0.73)	0.0005	rs4938723 diminishes NB risk. The stratified analysis demonstrates that rs4938723 TC/CC carriers are less prone to NB. Such association was found in both age subgroups, both sexes as well as all tumor sites and stages.	(141)

## Discussion

Recent studies have demonstrated abnormal expression of lncRNAs, miRNAs and circRNAs in neuroblastoma. Besides, some SNPs within lncRNAs and miRNAs confer risk of neuroblastoma. In vitro studies have shown the functional interactions between a number of these ncRNAs and MYCN, the oncogene that has essential roles in the pathogenesis of this type of cancer. Moreover, certain miRNAs have been shown to target tyrosine kinase receptors. For instance, hsa-miR-376c is predicted to target ALK tyrosine kinase receptor. Notably, this miRNA has been up-regulated in neuroblastoma samples of long-survivors (146). Expressions of a number of other ncRNAs have been shown to stratify neuroblastoma patients based on their risk of recurrence and clinical outcome.

The observed dysregulation of ncRNAs in neuroblastoma can be explained by their association with the frequent chromosomal abnormalities in this kind of cancer. Amplification of genomic loci corresponding to these transcripts is a possible route for their up-regulation (86). Moreover, epigenetic factors participate in the regulation of ncRNAs expression in neuroblastoma, as several lines of evidence points to the role of retinoic acid and its derivatives in the reversal of such dysregulation. Consistent with these observations, ATRA has been lately shown to induce differentiation of a number of neuroblastoma cell lines or activate apoptosis in these cells (147).

As a number of ncRNAs regulate tumorigenic process downstream of MYCN, dysregulation of these transcripts might represent an alternative mechanism of MYCN up-regulation/amplification in neuroblastoma. In vivo studies have demonstrated the efficacy of miRNA antagonism in suppression of proliferation of *MYCN*-amplified neuroblastoma cells in animal models (68). However, these results have not been replicated in clinical settings. Administration of miRNA mimics in clinical settings has encountered some problems most of the being related with the distribution of these transcripts in the body and enrichment in the target organs. Encapsulation of these small transcripts in nanoparticle vesicles is expected to enhance their stability and their presence in the circulation, permitting further time for their amassment in tumor tissues (148).

Multidrug resistance is a problem in the treatment of patients with neuroblastoma. Such phenotype has been associated with a number of genetic abnormalities such as over-expression of MYCN oncogene, hyper-activation of tyrosine kinase receptors (BDNF-TrkB) or reduced expression and activity of tumor suppressor genes including p53 (148). Therefore, ncRNAs that modulate expression of these elements or function in the downstream of these molecules can also be involved in the multidrug resistance of these cells. Therefore, modulation of expression of these transcripts represents a novel modality to combat multidrug resistance in neuroblastoma.

Expression profile of ncRNAs has been correlated with patients’ survival. The underlying mechanism of this observation has been clarified in some cases. For instance, hsa-miR-383, hsa-miR-548d-5p, hsa-miR-939 and hsa-miR-877* miRNAs which have been down-regulated in neuroblastoma samples from long-survivors (146) target a number of genes being involved in the neuronal differentiation (149).

Taken together, the above-mentioned evidence suggests the crucial roles of ncRNAs in the regulation of important aspects of cell survival, proliferation and differentiation and their participation in the pathogenesis of neuroblastoma. Their potential as therapeutic targets for this type of cancer should be more explored in the future studies. The main limitation of studies which assessed expression of ncRNAs in neuroblastoma is lack of longitudinal assessment of expression of these transcripts to unravel temporal changes during the course of disease. Conduction of this type of studies would facilitate approval of the diagnostic and prognostic power of ncRNAs.

## Author Contributions

MT and SG-F wrote the draft and revised it. OR, KHT, and MH performed the data collection, designed the tables and figures. All authors contributed to the article and approved the submitted version.

## Conflict of Interest

The authors declare that the research was conducted in the absence of any commercial or financial relationships that could be construed as a potential conflict of interest.
